# The impact of Zearalenone on heat-stressed skeletal muscle in pigs

**DOI:** 10.1093/jas/skac215

**Published:** 2022-08-01

**Authors:** Tori E Rudolph, Crystal M Roach, Lance H Baumgard, Jason W Ross, Aileen F Keating, Josh T Selsby

**Affiliations:** Department of Animal Science, Iowa State University, Ames, IA 50011, USA; Department of Animal Science, Iowa State University, Ames, IA 50011, USA; Department of Animal Science, Iowa State University, Ames, IA 50011, USA; Department of Animal Science, Iowa State University, Ames, IA 50011, USA; Department of Animal Science, Iowa State University, Ames, IA 50011, USA; Department of Animal Science, Iowa State University, Ames, IA 50011, USA

**Keywords:** glycolytic, heat shock proteins, hyperthermia, oxidative, oxidative stress

## Abstract

Heat stress (HS) and Zearalenone (ZEN) exposure affect growth, production efficiency, and animal welfare; and, under extreme situations, both can be lethal. Given that both HS and ZEN independently cause oxidative stress, we hypothesized that simultaneous exposure to HS and ZEN would cause greater oxidative stress in porcine skeletal muscle than either condition, alone. To address this hypothesis, crossbred, prepubertal gilts were treated with either vehicle control (cookie dough) or ZEN (40 μg/kg) and exposed to either thermoneutral (TN; 21.0 °C) or 12-h diurnal HS conditions (night: 32.2 °C; day: 35.0 °C) for 7 d. Pigs were euthanized immediately following the environmental challenge and the glycolytic (STW) and oxidative (STR) portions of the semitendinosus muscle were collected for analysis. In STR, malondialdehyde (MDA) concentration, a marker of oxidative stress, tended to increase following ZEN exposure (*P* = 0.08). HS increased CAT (*P* = 0.019) and SOD1 (*P* = 0.049) protein abundance, while ZEN decreased GPX1 protein abundance (*P* = 0.064) and activity (*P* = 0.036). In STR, HS did not alter protein expression of HSP27, HSP70, or HSP90. Conversely, in STW, MDA-modified proteins remained similar between all groups. Consistent with STR, ZEN decreased GPX1 (*P* = 0.046) protein abundance in STW. In STW, ZEN decreased protein abundance of HSP27 (*P* = 0.032) and pHSP27 (*P* = 0.0068), while HS increased protein expression of HSP70 (*P* = 0.04) and HSP90 (*P* = 0.041). These data suggest a muscle fiber type-specific response to HS or ZEN exposure, potentially rendering STR more susceptible to HS- and/or ZEN-induced oxidative stress, however, the combination of HS and ZEN did not augment oxidative stress.

## Teaser

Exposure to heat stress and mycotoxin is known to cause alterations in growth and production efficiency. Given the likelihood of simultaneous exposure, we examined how these pathological agents separately and collectively impact outcomes of muscle health in swine.

## Introduction

The impact of heat stress (HS) is multidimensional. HS affects growth, production efficiency, and overall animal health, and, under extreme situations, can result in death. Pigs may be more susceptible to HS compared with many species due to continued selection for increased lean tissue accretion, resulting in increased metabolic heat production (Brown-Brandl et al., 2014). Furthermore, pigs are unable to effectively reduce body temperature by sweating due to nonfunctional sweat glands (Baumgard and Rhoads, 2013). Even with mitigation strategies, HS continues to cost the U.S. pig industry approximately US$900 million annually (Baumgard and Rhoads, 2013). With environmental temperatures increasing globally, and more regular, extreme periods of heat and humidity, HS will continue to jeopardize animal health with increasing frequency and severity.

Zearalenone (ZEN) is a nonsteroidal estrogenic mycotoxin produced by fungi and can be present on a variety of livestock feeds such as corn and other grains, particularly during periods of high temperature and humidity (Uhlig et al., 2013), which are notably the same conditions that contribute to HS, raising the likelihood of concomitant HS and ZEN exposures. Pigs absorb approximately 85% of orally administered ZEN (Biehl et al., 1993), which is then converted into α-zearalenol. Conversely, cattle consuming ZEN largely convert it into β-zearalenol providing cattle some protection against ZEN-induced toxicity (Malekinejad et al., 2005) as α-zearalenol has higher estrogenicity and is metabolically more active than β-zearalenol (Keller et al., 2015). ZEN has been linked to reproductive dysfunction, and also negatively impacts a variety of systemic tissues (Shi et al., 2017; Cheng et al., 2020; Liu et al., 2020) and can even lead to immunosuppression and death (Young et al., 1990; Wang et al., 2014). In pig muscle, the anabolic effects of estrogens are mediated by decreased proteolysis (Rehfeldt et al., 2009) though ZEN appears to negatively impact muscle development through alterations in growth signaling molecules (Gao et al., 2016; Reddy et al., 2018). In cattle, estradiol is an anabolic growth hormone essential for the maintenance of the reproductive system and growth of skeletal muscle (Hunt et al., 1991) and α-zearalenol has been used to promote growth in cattle and sheep (Sinnett-Smith et al., 1983; Fumagalli et al., 1989).

Oxidative stress is a hallmark of environment-induced HS (Montilla et al., 2014; Cui and Gu, 2015; Volodina et al., 2017; Ganesan et al., 2018a; Houston et al., 2018) driven, at least in part, by mitochondrial dysfunction. Excess superoxide production frequently results from uncoupling of complexes I and III of the electron transport chain (Kikusato and Toyomizu, 2013; Huang et al., 2015), which has been observed following environmental HS (Kikusato and Toyomizu, 2013; Huang et al., 2015). Similarly, ZEN exposure promotes superoxide production, alters mitochondrial membrane potential, and induces mitochondrial swelling in a variety of tissues (Fan et al., 2017; Wang et al., 2018b; Xu et al., 2019); however, the impact of ZEN on porcine skeletal muscle, in vivo, remain largely unknown. Given that both HS and ZEN increase oxidative stress and the increased likelihood of ZEN contamination of feed during extreme heat events, we hypothesized simultaneous exposure to HS and ZEN would cause greater oxidative stress in porcine skeletal muscle than either condition, alone.

## Materials and Methods

### Animal treatments

All procedures were reviewed and approved by the Institutional Animal Care and Use Committee at Iowa State University (IACUC-19-147). To determine the extent to which ZEN exacerbates muscle injury caused by environment-induced HS, 4-mo old, crossbred, prepubertal gilts (*n* = 26; body weight [BW] 61.5 ± 0.5 kg) were housed individually in pens for a 3-d acclimation period. Following acclimation, all pigs remained in thermoneutral (TN) conditions and received cookie dough twice daily to familiarize to the treatment administration. Once adapted, one-half of the pigs were treated with cookie dough as vehicle control and the other half of the pigs received ZEN (20 μg/kg b.i.d.) per os in cookie dough at 0700 and 1900 hours. Treatment with vehicle control or ZEN began at the onset of environmental treatment and continued throughout the treatment period. Approximately one-half of placebo-treated pigs and one-half of ZEN-treated pigs were randomly assigned to TN conditions (21.0 ± 0.10 °C, 66.8% relative humidity) or 7-d, 12-h diurnal HS conditions (night: 32.2 ± 0.1 °C, 40.7% relative humidity; day: 35.0 ± 0.2 °C, 42.0% relative humidity) such that there were four groups: TN control (*n* = 6), HS control (*n* = 7), TN ZEN (*n* = 6), HS ZEN (*n* = 7). All animals were exposed to a 12:12 h light–dark cycle and given ad libitum access to a standard grow-finisher diet (Table 1) and water for the duration of the experiment. Rectal temperature and respiration rate were monitored and recorded at 0700 and 1900 hours. Respiration rates were collected by counting flank movement for 15 s and multiplying the given value by 4 to obtain breaths per minute (bpm). Following the treatment period, animals were sacrificed by captive bolt and exsanguination. The glycolytic (STW) and oxidative (STR) portions of the semitendinosus muscle were collected and frozen in liquid nitrogen for subsequent analyses.

**Table 1.** Diet composition

Ingredient	%
Corn	61.16
Soybean meal	16.40
DDGs^1^	20.00
Vitamin-mineral Premix SCE 45-30	2.00
Lysine	0.32
Threonine	0.0095
Calcium	0.1

Distilled dried grain with soluble.

### Protein extraction and western blot

Protein was extracted and western blotting was performed as previously described (Rudolph et al., 2021). Briefly, frozen muscle samples were powdered on dry ice and homogenized in protein extraction buffer (10 mmol/L sodium phosphate and 2% SDS, pH 7.0) containing protease inhibitor (Halt protease inhibitor cocktail, ThermoFisher Scientific, Inc.), centrifuged (1,500 × *g*, 10 min, 4 °C), and the supernatant collected. Protein concentrations were determined using the Pierce BCA Protein Assay (ThermoFisher Scientific, Inc.). Samples were loaded onto precast polyacrylamide gels (Bio-Rad, Hercules, CA, USA or Genscript, Piscataway, NJ, USA), separated by molecular mass, and transferred onto nitrocellulose membranes. To ensure equal sample loading, membranes were stained with Ponceau S stain and imaged using the Azure Biosystems c600 imaging system. The total optical density of each lane was objectively quantified and was similar for all groups on all membranes. Membranes were washed in TBST (50 mmol/L Tris–HCl, 150 mmol/L NaCl, 0.1% Tween-20, pH 7.4) to remove the Ponceau S, blocked in 5% dehydrated fat-free milk dissolved in TBST for 1 h at room temperature, and incubated in primary antibody overnight at 4 °C. Primary and secondary antibodies were suspended in TBST (unless otherwise noted) and diluted as follows: 4-hydroxynonenal (4-HNE) (Abcam, ab48506, primary 1:1,000 1% milk, secondary 1:4,000), catalase (CAT) (Cell Signaling Technology (CST), #14097, primary 1:1,000, secondary 1:2,000 2% dehydrated milk TBST solution), Cu/Zn superoxide dismutase (SOD1) (Abcam, ab13498, primary 1:1,000, secondary 1:3,000), glutathione peroxidase 1 (GPX1) (Abcam, ab22604, primary 1:1,000, secondary 1:2,000), Heat Shock Protein 27 (HSP27) (Abcam, ab2790, primary 1:1,000, secondary 1:2,000), phospho-HSP27 (Ser82) (CST, #2406, primary 1:1,000, secondary 1:2,000), Heat Shock Protein 70 (HSP70) (Novus Biologicals, NB110-96427, primary 1:1,000, secondary 1:2,000 2.5% milk), Heat Shock Protein 90 (HSP90) (CST, #4877, primary 1:1,000, secondary 1:2,000), malondialdehyde (MDA) (Abcam, ab27642, primary 1:5,000 1% milk, secondary 1:2,000), Mn-superoxide dismutase (SOD2) (Abcam, ab13533, primary 1:3,000 2.5% milk, secondary 1:4,000 5% milk).

After incubation overnight, membranes were washed in TBST, then rocked in secondary antibody for 1 h, and washed again in TBST. Membranes were exposed to enhanced chemiluminescence (Bio-Rad) and protein bands were captured using the Azure Biosystems c600 imaging system. The detected bands were quantified using the AzureSpot Software using automated band detection where possible to limit bias.

### mRNA isolation

mRNA was isolated from approximately 25 mg of powdered muscle tissue via Trizol extraction (20:1 volume:weight ratio). Following extraction, 200 μL chloroform was added to the microcentrifuge tube, vigorously shaken, and then incubated for 3 min at room temperature. Samples were centrifuged at 12,000 × *g* for 15 min at 4 °C. The aqueous phase (upper phase) was removed, placed into a new microcentrifuge tube, and combined with an equal volume of isopropanol. Tubes were inverted several times, incubated at room temperature for 10 min, and then centrifuged for 10 min at 12,000 × *g* at 4 °C. The supernatant was discarded, and the pellet was left to air dry for 30 min. The pellet containing the RNA was washed by vortexing in 500 μL of 75% EtOH and centrifuged at 7,500 × *g* for 5 min at 4 °C. The pellet was solubilized in 25 μL of RNase-free water by incubating for 10 min at 55 °C. RNA concentration and purity were determined using a Nanodrop Spectrophotometer and measuring absorbance at 260/280 nm.

### Real-time quantitative polymerase chain reaction

cDNA synthesis was performed using the Verso cDNA synthesis kit according to manufacturer instructions (ThermoFisher Scientific, Inc.). Following cDNA synthesis, 80 μL of water was added to all samples, for a final cDNA concentration of 10 ng/uL. Relative transcript abundance was measured using QuantiFast SYBR Green PCR Kit (Qiagen, Valencia, CA). Real-time qPCR primer sequences are provided in Table 2. Briefly, reactions consisted of 10 µL of SYBR Green PCR master mix, 2 µL of forward primer and 2 µL of reverse primer (Table 2) at a concentration of 4 mM, 1 µL of template DNA (10 ng/μL), and 4 µL of water for a total volume of 20 µL. qPCR plates were placed into a thermocycler for amplification using cycling parameters of 95 °C for 5 min, 95 °C for 15 s for denaturation, 30 s at 60 °C for annealing, and elongation for 40 cycles. Melting curve analysis was performed and visually inspected to confirm amplification of a single product. In addition, no-template controls were included to ensure no reaction contamination. All statistical analyses were performed using the ΔCT values with 18S as the control transcript, and resultant data were reported as fold changes relative to TN control.

**Table 2.** qRT-PCR primer sequences

Target gene	Forward primer	Reverse primer
18S rRNA	AAACGGCTACCACATCCAAG	TCGCGGAAGGATTTAAAGTG
*CAT*	CAGCTTTAGTGCTCCCGAAC	AGATGACCCGCAATGTTCTC
*GPX1*	GATGAATGAGCTGCAGCGG	CCATTCACCTCACACTTC
*SOD1*	GAGACCTGGGCAATGTGACT	CCAAACGACTTCGAGCATTT
*SOD2*	CGCTGAAAAAGGGTGATGTT	AGCGGTCAACTTCTCCTTGA

### Redox balance

Malondialdehyde (MDA) is a product of lipid peroxidation. The thiobarbituric acid-reactive substances (TBARS) assay was used to measure MDA-TBA adduct formation following the manufacturer’s instruction (Cayman cat. No 700870). Briefly, approximately 25 mg of muscle tissue was weighed and homogenized in whole muscle buffer, centrifuged at 1,600 × *g* for 10 min at 4 °C and the supernatant was collected and used for analysis. MDA-TBA adduct formation was measured colorimetrically at 530 nm using a microplate reader (BioTek, Winooski, VT) and is expressed in µM.

Protein carbonyl content is a common marker used to identify protein oxidation. Protein carbonyl concentration was determined spectrophotometrically using the Cayman Protein Carbonyl Colorimetric Assay Kit (cat. No 10005020) according to the manufacturer’s instructions. Briefly, muscle samples were homogenized in phosphate buffer (50 mM sodium phosphate, 1 mM EDTA, pH 7.0), centrifuged for 15 min at 10,000 × *g* at 4 °C, supernatant collected, and protein concentration determined. All samples were then normalized to a protein concentration of 4 mg/mL. This assay utilizes the DNPH reaction with protein carbonyls to measure the protein carbonyl content in samples. Protein carbonyl content is expressed in nmol/mg.

Glutathione (GSH) is a non-protein thiol capable of preventing damage caused by reactive oxygen species. The Invitrogen Glutathione Colorimetric Detection Kit (cat. No EIAGSHC) uses a colorimetric substrate that reacts with GSH and oxidized glutathione (GSSG) content in samples. To measure GSH and GSSH 10 mg of muscle tissue was homogenized in 250 μL of 100 mM phosphate buffer, pH 7.0. Samples were deproteinated with 5% sulfosalicylic acid dihydrate (SSA) and either left untreated (for measurement of GSH) or treated with 4-vinylpyrimidine (for GSSG measurement). Absorbance was measured at 405 nm. GSH and GSSG concentrations are expressed in μM/mg/mL protein. The ratio of GSSG/GSH was calculated by dividing the measured GSSG concentration by the measured GSH concentration.

### Enzymatic activities

Total SOD activity was assessed using a Cayman Chemical’s Superoxide Dismutase Assay Kit (cat. No 706002) with activity measured colorimetrically from muscle tissue homogenate (20 mM HEPES buffer, 1 mM EGTA, 210 mM mannitol, 70 mM sucrose, pH 7.2). This assay utilizes tetrazolium salt for the detection of superoxide radicals generated by xanthine oxidase and hypoxanthine. Following a 30-min room temperature incubation, absorbance at 440 nm was determined using a microplate reader (Bio-Tek, Winooski, VT). Total SOD activity was expressed as U/mL/mg of protein. CAT activity (nmol/min/mL) was measured using a Catalase Assay Kit (Cayman Chemical, cat. No 707002) according to the manufacturer’s instructions. Tissue was homogenized in phosphate buffer (50 mM sodium phosphate, 1 mM EDTA, pH 7.0). In this approach, catalase reacts with methanol in the presence of H_2_O_2_ to create formaldehyde, which is then measured at a single time point spectrophotometrically at 540 nm. Glutathione peroxidase (GPX) activity (μmol/min/mL) was measured via Cayman Chemical’s Glutathione Peroxidase Assay Kit (cat. No 703102). The assay measures GPX activity indirectly by a coupled reaction with glutathione reductase (GR). Oxidized glutathione (GSSG) is produced upon reduction of hydroperoxide by GPX and is then recycled to its reduced (GSH) state by GR and NADPH. The oxidation of NADPH to NADP+ results in decreased absorbance and the rate of decreased absorbance at 340 nm are directly proportional to GPX activity in the sample.

### Statistical analysis

Data were compared using a 2 × 2 ANOVA with the main effects of HS and ZEN treatment along with an HS × ZEN interaction. A Newman–Keuls post hoc test was performed where appropriate. Data are presented as means ± SEM. Statistical significance was considered as *P* < 0.05. A trend for biologically meaningful differences was considered if 0.05 < *P* ≤ 0.10.

## Results

### Phenotypic parameters

To successfully induce HS, gilts were subjected to a 7-d cyclic heating protocol. Briefly, 7 d of HS elevated rectal temperature in both morning (TN: 38.9 ± 0.15 °C; HS: 39.3 ± 0.14; *P* < 0.001) and night (TN: 39.2 ± 0.10 °C; HS: 39.8 ± 0.09 °C; *P* < 0.0001) measures. ZEN caused a reduction in rectal temperature compared to the HS Control both morning (HS Control: 39.50 ± 0.14 °C; HS ZEN: 39.1 ± 0.14 °C; *P* = 0.047) and night (HS Control: 40.0 ± 0.09 °C; HS ZEN: 39.7 ± 0.09 °C; *P* = 0.037). Similarly, 7 d of HS increased respiration rate compared to TN in both morning (71 vs. 37 bpm; *P* < 0.0001) and night (87 vs. 39 bpm; *P* < 0.0001). There was no significant effect of ZEN on respiratory rate.

### Oxidative muscle

**Figure 1.**
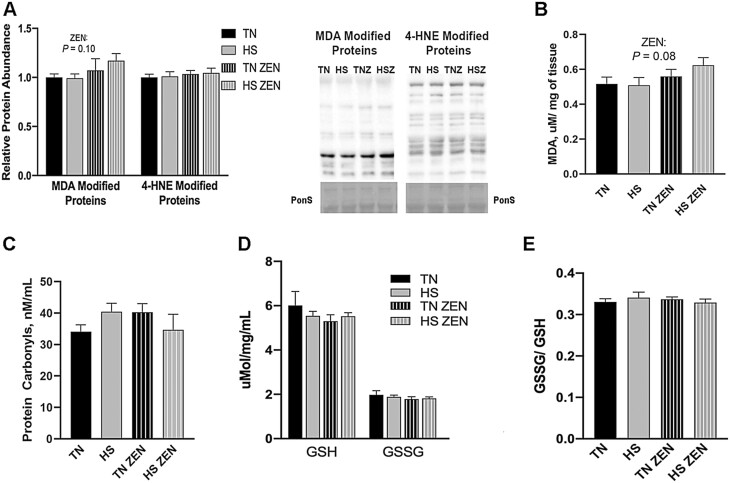
Assessment of redox status following 7 d of diurnal heat stress (HS) in oxidative skeletal muscle. Oxidative stress was measured by quantifying the relative protein abundence of malondialdehyde (MDA)- and 4-hydroxynonenol (HNE)-modified proteins in Semitendinosus red (STR). To assess oxidative modification of lipids (A) MDA- and 4-HNE-modified proteins were assessed via western blotting. (B) MDA (TBARS) concentration was determined colorimetrically and expressed in µM/mg of tissue. (C) The concentration of protein carbonyls (nmol/mL) was measured to assess oxidative modification of protein. We also considered (D) the concentration of reduced (GSH), oxidized (GSSG) glutathione expressed in μmol/mg/mL, and (E) the ratio of GSSH/GSH. Values represent the mean ± SEM. Groups (*n* = 6–7/group) were compared using a 2 × 2 ANOVA. A main effect of zearalenone (ZEN) (*P* < 0.05) is indicated.

To determine the extent to which 7 d of cyclic HS with or without ZEN treatment increased lipid oxidation, relative abundance of MDA and 4-HNE-modified proteins and concentration of MDA were measured (Figure 1A and B). Heat stress and/or ZEN did not increase the relative protein abundance of 4-HNE-modified proteins; however, MDA-modified proteins (*P* = 0.10) and MDA concentration (*P* = 0.08) tended to be increased by 20% by ZEN, largely driven by the HS-ZEN group. To further assess oxidative stress, protein carbonyl concentration, an indicator of protein oxidation, was determined. Treatment with ZEN and/or environmental HS did not alter protein carbonyl concentration (Figure 1C). GSH and GSSG concentration (Figure 1D) and the ratio of GSSG/GSH were also similar between groups (Figure 1E).

**Figure 2.**
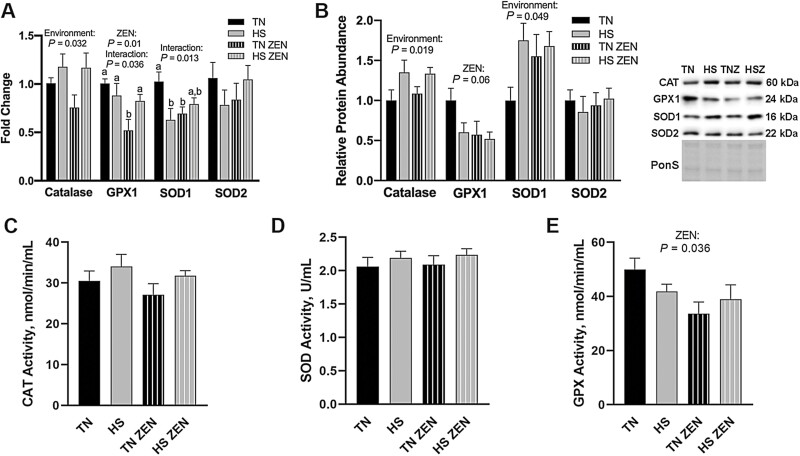
Assessment of antioxidant enzymes following 7d of diurnal HS in oxidative skeletal muscle. (A) Transcript expression and (B) relative protein abundance of antioxidant enzymes following HS. Ponceau S stain (PonS) was used as a loading control. (C) Enzymatic activity of Catalase (CAT; nmol/min/mL), (D) total superoxide dismutase (SOD; U/mL), and (E) glutathione peroxidase (GPX; nmol/min/mL) were measured. Groups were compared using a 2 × 2 ANOVA; (*n* = 6–7/group). A main effect of environment (*P* < 0.05) and/or a main effect of ZEN (*P* < 0.05) are indicated. Values represent the mean ± SEM. Similar groups are indicated by the same letter where appropriate, with differences determined by a Newman–Keuls post hoc test.

Antioxidant enzymes play an important role in maintaining and restoring redox balance. *CAT* transcript expression was increased 0.1-fold in HS compared with TN animals (*P* = 0.032). *GPX1* mRNA decreased 0.4-fold as a main effect of ZEN exposure (*P* = 0.010) and an interaction was discovered such that *GPX1* was increased in HS-ZEN animals compared to TN-ZEN (*P =* 0.036) (Figure 2A). Relative transcript abundance of *SOD1* was decreased in HS-Con and TN-ZEN groups compared to TN-Con; however, an HS × ZEN interaction (*P* = 0.013) was observed such that *SOD1* in HS-ZEN was elevated to be statistically similar to TN-Con. Relative transcript expression of SOD2 was similar between groups (Figure 2A). Protein abundance of CAT (*P* = 0.019) and SOD1 (*P* = 0.049) increased 0.25- and 0.6-fold, respectively, as a main effect of HS, while GPX1 tended to decrease as a main effect of ZEN (*P* = 0.064) and SOD2 protein abundance were similar among groups (Figure 2B). Catalase enzyme activity was similar among groups (*P* = 0.12; Figure 2C) as was total SOD activity (Figure 2D). Treatment with ZEN, however, decreased GPX activity by 0.2-fold compared to vehicle-treated animals (*P* = 0.036) (Figure 2E).

**Figure 3.**
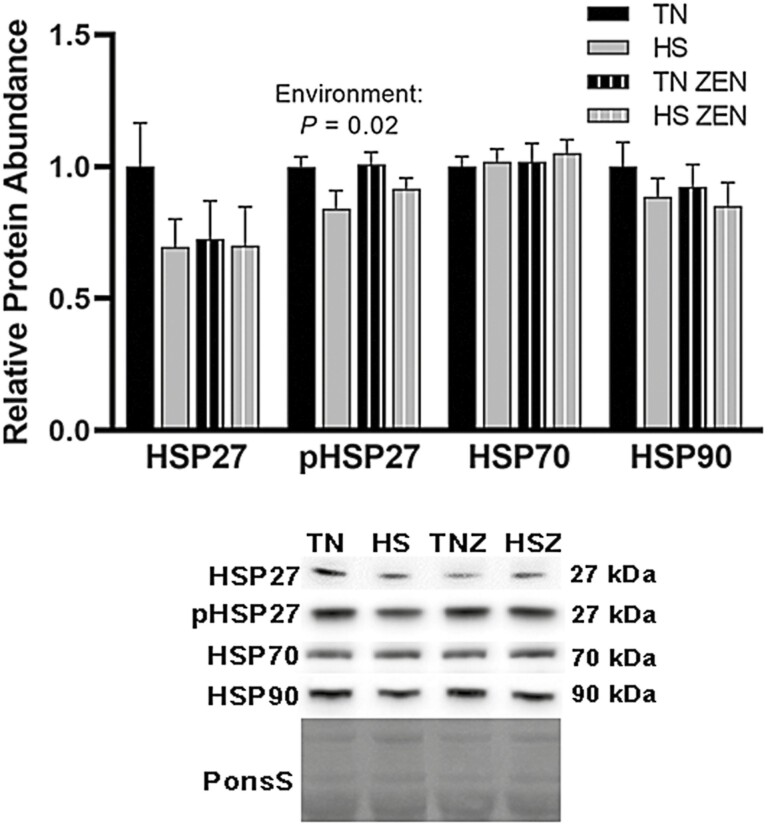
Assessment of heat shock proteins (HSPs) following 7 d of diurnal heat stress (HS) in oxidative skeletal muscle. Relative protein abundance was measured via western blotting and quantified (*n* = 6–7/group). Ponceau S Stain (PonS) was used as a loading control. Values represent the mean ± SEM. Groups were compared using a 2 × 2 ANOVA. A main effect of environment (*P* < 0.05) is indicated.

Heat shock proteins (HSP) maintain proteostasis and protect cells from dysfunction and/or death during various stress conditions, including heat (Liu and Steinacker, 2001). Given this, protein abundance of HSP27, p-HSP27, HSP70, and HSP90, all of which are expressed in skeletal muscle (Liu and Steinacker, 2001), was quantified. Protein abundance of p-HSP27 (Ser82) decreased 0.20-fold as a main effect of HS (*P* = 0.021); all other HSPs remained similar among groups (Figure 3).

### Glycolytic muscle

**Figure 4.**
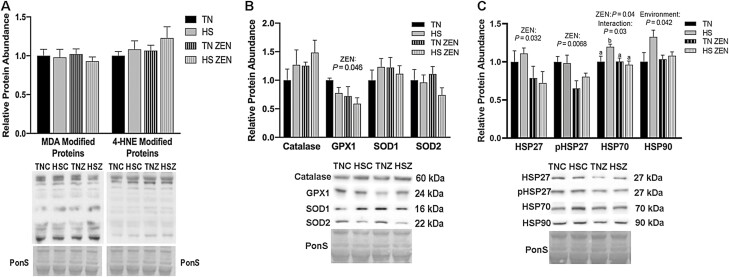
Effects of 7-d dirual HS and ZEN treatment on glycolytic skeletal muscle. (A) After 7 d of HS, relative protein abundance of MDA- and 4-HNE-modified proteins was similar between all groups. (B) Antioxidant enzymes relative protein abundance were assessed via western blotting. Treatment with ZEN decreased protein abundance in GPX1; however, all other antioxidant enzymes were similar between groups (*n* = 6–7/group). (C) Relative protein abundance of select HSPs following environmental HS and ZEN exposure. Ponceau S Stain (PonS) was used as a loading control. Values represent the mean ± SEM. Groups were compared using a 2 × 2 ANOVA; a main effect of environment *(P* < 0.05) and/or a main effect of zearalenone (ZEN) (*P* < 0.05) is indicated. Similar groups are indicated by the same letter where appropriate, with differences determined by a Newman–Keuls post hoc test.

To investigate the extent to which glycolytic muscle responds to ZEN exposure and/or 7-d diurnal HS, oxidative stress, antioxidant abundance, and HSP abundance were measured in STW. Relative abundance of MDA- and 4-HNE-modified proteins was similar between groups (Figure 4A). Additionally, relative protein abundance of the antioxidants CAT, SOD1, and SOD2 were similar between all groups, while GPX1 was decreased by 0.4-fold as a main effect of ZEN treatment (*P* = 0.046) (Figure 4B).

In a notable contrast to STR, in the STW HS and ZEN caused a variety of changes in the relative protein abundance of HSPs. Treatment with ZEN caused a 0.3-fold reduction in HSP27 (*P* = 0.032) as well as a 0.25-fold reduction in p-HSP27 (Ser82) (*P* = 0.0068) compared to vehicle-treated animals. We detected an interaction (*P* = 0.033) between HS and ZEN exposure on HSP70 protein abundance such that an HS-mediated elevation in HSP70 level was blunted by ZEN exposure (*P* = 0.04). Additionally, HSP90 expression increased as a main effect of HS (*P =* 0.041; Figure 4C).

## Discussion

Both HS and ZEN exposure pose a threat to animal and human health. Pigs are at an increased risk of HS as they do not have functional sweat glands (Baumgard and Rhoads, 2013) and selective breeding for increased growth rate has caused increased metabolic heat production (Brown-Brandl et al., 2014). Similarly, ZEN causes estrogenic effects in a variety of livestock, including pigs (Malekinejad et al., 2005). Data regarding ZEN exposure and human health are limited; however, ZEN may cause hyperestrogenic syndrome and early puberty in women and children (Massart et al., 2008). Studies in animals document hepatotoxicity (Gao et al., 2018), hematotoxicity (Yang et al., 2016), renal toxicity (Wang et al., 2018a), disruption of blood coagulation (Maaroufi et al., 1996), and reproductive disorders, which can ultimately result in infertility (Zinedine et al., 2007). Both HS and ZEN exposure have been linked to oxidative stress in a variety of tissues in vitro and in vivo (Mujahid et al., 2009; Jiang et al., 2011; Montilla et al., 2014; Fan et al., 2017; Ganesan et al., 2018a), but the impact of ZEN exposure alone or in combination with HS in skeletal muscle, in vivo, remain largely unknown. We hypothesized simultaneous exposure to HS and ZEN would cause greater oxidative stress in porcine skeletal muscle than either condition, alone.

In total, in oxidative muscle, lipid oxidation tended to increase following 7 d of diurnal HS with ZEN exposure. This outcome is consistent with previous work as both HS and ZEN cause oxidative stress in vitro (Lee et al., 2013; Fan et al., 2017; Alemu et al., 2018) and in vivo (Montilla et al., 2014; Volodina et al., 2017; Ganesan et al., 2018a). Lipids are potentially more susceptible to oxidative modification than proteins in this experimental approach. Selective modification of lipid while sparing protein is consistent with our previous findings in HS skeletal muscle (Volodina et al., 2017). In the present experiment, HS increased transcript expression and protein abundance of CAT, which may protect against oxidative stress. Conversely, ZEN, alone, interferes with the antioxidant response of cells and suppresses the abundance and activities of SOD and GPX (Fan et al., 2017; Cheng et al., 2020; Liu et al., 2020). Superoxide Dismutase converts superoxide (O_2_·^**–**^) into hydrogen peroxide (H_2_O_2_) and GPX is responsible for the reduction of H_2_O_2_ to H_2_O to prevent cellular toxicity. Failure to reduce H_2_O_2_ to H_2_O can result in the production of the dangerous ·OH radical, which can react with and damage cellular molecules. Indeed, ZEN contamination decreased both total SOD and GPX antioxidant activities in post-weaning gilt duodenum and liver, and ultimately led to oxidative stress (Shi et al., 2017; Cheng et al., 2020). Consistent with these observations, SOD1 protein abundance was increased without a corresponding change in enzymatic activity, which could be the result of protein oxidation (Lin et al., 2019). Additionally, we observed decreased *GPX1* mRNA level, protein abundance, and decreased GPX enzymatic activity in oxidative skeletal muscle. The reduction in GPX enzymatic activity with ZEN exposure was reflected in the increased MDA-modified protein abundance and MDA concentration.

One of the many roles of HSPs is to protect cells from oxidative stress during a variety of stresses such as HS and mitochondrial dysfunction (Gomez-Pastor et al., 2018). ZEN may cause mitochondrial dysfunction by altering calcium transport, mitochondrial membrane potential, and inducing mitochondrial swelling (Wu et al., 2020). In the present study, HSP70 abundance increased following HS in glycolytic muscle but remained similar between groups in oxidative muscle. Differential responses of HSPs have been reported between muscle fiber types (Oishi et al., 2002, 2003; Cruzen et al., 2015); however, the specific mechanism causing this modified response remains unknown. A failed HSP response is counterintuitive as HSPs are commonly upregulated following HS in a variety of tissues (Pearce et al., 2014, 2015; Xie et al., 2014); however, we have reported that skeletal muscle remains largely resistant to increased HSP following HS (Ganesan et al., 2016, 2017; Brownstein et al., 2017). In this study, ZEN decreased HSP27, p-HSP27 (Ser82), and HSP70 abundance in STW, though did not alter HSP expression in STR. This is surprising as ZEN has upregulated HSP27 and HSP70 expression in liver cells, in vitro (Hassen et al., 2005; El Golli Bennour et al., 2009; Lee et al., 2013), and small intestine, in vivo (Liu et al., 2020). Taken together, these data suggest a tissue-specific response of HSPs to HS and ZEN exposure, potentially leaving STR more susceptible to HS- and/or ZEN-induced oxidative stress. Reductions in HSP27 and HSP70 protein abundance induce autophagy and apoptosis, respectively (Kim et al., 2016), which play important roles in maintaining cellular homeostasis (Mitter et al., 2014; Wang et al., 2014). We have demonstrated that increased degradation of autophagosomes coincided with a decrease in MDA-modified protein abundance following HS (Volodina et al., 2017; Ganesan et al., 2018b); which could partially explain why oxidative stress in glycolytic muscle was not observed but occurred in oxidative muscle in this study.

ZEN occurs primarily on wheat and corn across the United States at concentrations of 0.36–11.05 and 0.114–3 mg/kg feed, respectively (Zinedine et al., 2007). In this experiment, pigs were fed a ZEN dose of 0.04 mg/kg BW/, which has been identified as the no observed adverse effect level (NOAEL) in mature female pigs (Knutsen et al., 2017). Based on the final BW, pigs received between 2.44 and 2.48 mg ZEN/d. Other studies measuring ZEN toxicity in pigs used between 0.20 and 1.6 mg/kg BW/d delivered orally (Obremski et al., 2003; Zwierzchowski et al., 2005; Wu et al., 2021) or 0.1–10 mg/kg of diet fed (Jiang et al., 2011; Su et al., 2018; Wu et al., 2020). As the physiological impact and relationship between ZEN and HS have not been empirically established, we utilized a low dose of ZEN and mild HS conditions. Despite the mild HS, rectal temperatures were elevated throughout the experimental period in both HS groups compared to the TN controls, indicating that we successfully implemented HS.

In conclusion, ZEN exposure tended to increase oxidative stress in oxidative skeletal muscle but not glycolytic muscle in exposed prepubertal gilts. This finding adds additional insight to previous work indicating estrogen failed to support increased growth in swine (Roche and Quirke, 1986; Rehfeldt et al., 2007). The elevated MDA-modified proteins and MDA concentration appear to be largely driven by the HS-ZEN group following low dose ZEN and a mild heat stress. Consistent with previously published reports, ZEN appeared to negatively regulate GPX transcript and protein abundance, and enzyme activity as well as decrease HSP expression. Higher environmental temperatures and a larger ZEN dose could further exacerbate oxidative stress by further reductions in antioxidant enzymatic activity and HSP abundance, both of which are responsible for maintaining cellular redox balance.

## Abbreviations

BW: body weight
CAT: catalase
GPX1: glutathione peroxidase 1
GSH: reduced glutathione
GSSG: oxidized glutathione
HS: heat stress
HSP: Heat Shock Protein MDA, malondialdehyde
PonS: ponceau S
SOD: superoxide dismutase
STR: semitendinosus red
STW: semitendinosus white
TN: thermoneutral
Zen: zearalenone
4-HNE: 4-hydroxynonenol
